# Isolating an active and inactive CACTA transposon from lettuce color mutants and characterizing their family

**DOI:** 10.1093/plphys/kiab143

**Published:** 2021-03-26

**Authors:** Csanad Gurdon, Alexander Kozik, Rong Tao, Alexander Poulev, Isabel Armas, Richard W Michelmore, Ilya Raskin

**Affiliations:** 1 Department of Plant Biology, Rutgers University, New Brunswick, New Jersey 08901-8520, USA; 2 UC Davis Genome Center, Davis, California 95616, USA

## Abstract

Dietary flavonoids play an important role in human nutrition and health. Flavonoid biosynthesis genes have recently been identified in lettuce (*Lactuca sativa*); however, few mutants have been characterized. We now report the causative mutations in Green Super Lettuce (GSL), a natural light green mutant derived from red cultivar NAR; and GSL-Dark Green (GSL-DG), an olive-green natural derivative of GSL. GSL harbors *CACTA 1* (*LsC1*), a 3.9-kb active nonautonomous CACTA superfamily transposon inserted in the 5′ untranslated region of *anthocyanidin synthase* (*ANS*), a gene coding for a key enzyme in anthocyanin biosynthesis. Both terminal inverted repeats (TIRs) of this transposon were intact, enabling somatic excision of the mobile element, which led to the restoration of *ANS* expression and the accumulation of red anthocyanins in sectors on otherwise green leaves. GSL-DG harbors *CACTA 2* (*LsC2*), a 1.1-kb truncated copy of *LsC1* that lacks one of the TIRs, rendering the transposon inactive. RNA-sequencing and reverse transcription quantitative PCR of NAR, GSL, and GSL-DG indicated the relative expression level of *ANS* was strongly influenced by the transposon insertions. Analysis of flavonoid content indicated leaf cyanidin levels correlated positively with *ANS* expression. Bioinformatic analysis of the *cv* Salinas lettuce reference genome led to the discovery and characterization of an *LsC1* transposon family with a putative transposon copy number greater than 1,700. Homologs of *tnpA* and *tnpD*, the genes encoding two proteins necessary for activation of transposition of CACTA elements, were also identified in the lettuce genome.

## Teaser

Isolation of active and inactive alleles of a CACTA superfamily transposon from an anthocyanin biosynthesis gene in lettuce led to characterization of a transposon family of >1,700 copies.

## Introduction

With a consumption of 11.2 kg per capita, lettuce (*Lactuca sativa* L.) is the third most commonly consumed vegetable in the USA. In 2018, the USA produced over 3.6 billion kg of lettuce, worth $1.9 billion (Parr et al., 2019). Most lettuce varieties are the good source of fiber, iron, folic acid, and vitamin C (Kim et al., 2016; Simko, 2019). In addition, some cultivars contain high levels of phenolic compounds, phytonutrients that have been associated with beneficial health outcomes (for review, see Crozier et al., 2009). The most ubiquitous phenolic compounds in lettuce are phenolic acids, predominantly caffeic acid derivatives, and flavonol glycosides, chiefly quercetin 3-O-malonylglucoside, quercetin 3-O-glucoside, and quercetin 3-O-glucuronide (Ferreres et al., 1997; Llorach et al., 2008; Damerum et al., 2015; Kitazaki et al., 2018). In addition, red lettuces accumulate the anthocyanin cyanidin 3-O-malonylglucoside, which green lettuces lack (Ferreres et al., 1997; Llorach et al., 2008; Kitazaki et al., 2018). Although most lettuce cultivars contain the same flavonoids and phenolic acids, their level varies greatly in the lettuce germplasm: red leaf lettuce cultivars have the highest concentration of total phenolics and flavonoids, while green crisphead cultivars commonly consumed in the USA have low levels (Llorach et al., 2008; Kim et al., 2016; van Treuren et al., 2018; Simko, 2019).

Vegetables with high levels of phenolic compounds are considered desirable for their perceived health benefit (Tome-Carneiro and Visioli, 2016; Zhang and Tsao, 2016). Thus, efforts have been focused on the development of lettuce cultivars with enhanced nutritional or functional value (Cheng et al., 2014; Damerum et al., 2015; Gurdon et al., 2019) and the characterization of key enzyme and regulatory genes of flavonoid biosynthesis. In lettuce, some genes of the flavonoid biosynthesis pathway were identified by Park et al. (2007) and Zhang et al. (2016) based on homology to well-characterized genes from other species (for reviews on flavonoid biosynthesis, see Shirley et al., 1995; Winkel-Shirley, 2001; Bowerman et al., 2012; Saito et al., 2013; Appelhagen et al., 2014; Shi and Xie, 2014). The assembly of a high quality, annotated lettuce genome (Reyes-Chin-Wo et al., 2017) allowed for the identification of 153 genes associated with flavonoid biosynthesis in the species (Zhang et al., 2017). This was achieved by RNA-sequencing 240 diverse accessions followed by expressed Quantitative Trait Locus (eQTL) mapping in 180 lines, homology-based gene annotation, and coexpression analysis (Zhang et al., 2017). In addition to the 153 flavonoid biosynthesis-associated genes, Zhang et al. (2017) identified 80 transcripts that were coexpressed with at least 3 of 18 key enzymes or regulator genes of the pathway, indicating these 80 genes potentially interact with or play a role in flavonoid biosynthesis. Recently, Su et al. (2020) mapped and characterized four transcription factors controlling red leaf color in lettuce.

The Raskin laboratory established a research program to develop lettuce lines high in different flavonoids. Within this program, tissue culture selection for deep purple color, an indicator of high anthocyanin content, was utilized to develop three deep red Rutgers Scarlet Lettuce (RSL) lines (NAR, NBR, and NFR) from existing red cultivars (Cheng et al., 2014). RSL lines had high levels of anthocyanins and total phenolics: more than 2% and 9% dry weight, respectively (Cheng et al., 2014). After multiple generations of maintaining lines by self-pollination, a spontaneous green revertant, later named Green Super Lettuce (GSL), was detected among red seedlings of NAR, an RSL line derived from *cv* Annapolis (Armas Gutierrez, 2015). GSL had light green leaves with irregular red sectors in them, indicating the phenotype was likely caused by an active DNA transposon and that the green phenotype was recessive (Armas Gutierrez, 2015). A limited biochemical analysis suggested that GSL almost entirely lacked anthocyanins, but it accumulated much higher levels of quercetin glycosides than NAR (Armas Gutierrez, 2015). Total phenolic content in NAR and GSL was not statistically different when grown under identical circumstances (Armas Gutierrez, 2015). Over the past 5 years, no further green revertants have been discovered among hundreds of RSL seedlings, indicating that the spontaneous mutation responsible for the GSL phenotype was likely stochastic and rare.

GSL plants did not segregate for color; however, a spontaneous mutant was found in the Raskin lab among GSL seedlings after multiple generations of seed propagation. This plant had dark olive-green leaves with red sectors and segregated to three offspring phenotypes: (1) GSL (light green, with red spots); (2) GSL-Dark Green (GSL-DG, dark olive green with no red leaf sectors); and (3) GSL-DG with red leaf sectors (GSL-DG(r)). This indicated that GSL-DG lettuce harbored a second allele of the locus responsible for the GSL phenotype. The pedigree of all lines investigated in this report is shown in Supplemental Figure S1.

Here we report the comprehensive molecular and phytochemical analysis of NAR, GSL, and GSL-DG based on RNA-sequencing (RNA-seq) and reverse transcription quantitative PCR (RT-qPCR) validation of RNA-seq results, as well as the quantification of major flavonoids in all lines by ultra-performance liquid chromatography–tandem mass spectrometry (UPLC–MS/MS). We report that GSL harbors the nonautonomous CACTA superfamily transposon *Lactuca sativa CACTA 1 (LsC1*), a 3.9-kb mobile DNA element inserted in the 5′ untranslated region (UTR) of *anthocyanidin synthase* (*ANS*), a gene coding for a key enzyme in anthocyanin biosynthesis. Intact terminal inverted repeats (TIRs) of this transposon enable its somatic excision, which leads to restoration of *ANS* expression and accumulation of red anthocyanins in sectors on otherwise green leaves. GSL-DG harbors *LsC2*, a 1.1 kb truncated copy of *LsC1* that lacks one of the TIRs, rendering the transposon inactive. Additionally, we thoroughly characterized the transposon family containing *LsC1* and *LsC2*, utilizing the published lettuce genome (Reyes-Chin-Wo et al., 2017) and our data.

## Results

### GSL and GSL-DG harbor different alleles of the same transposon insertion

**Figure 1:**
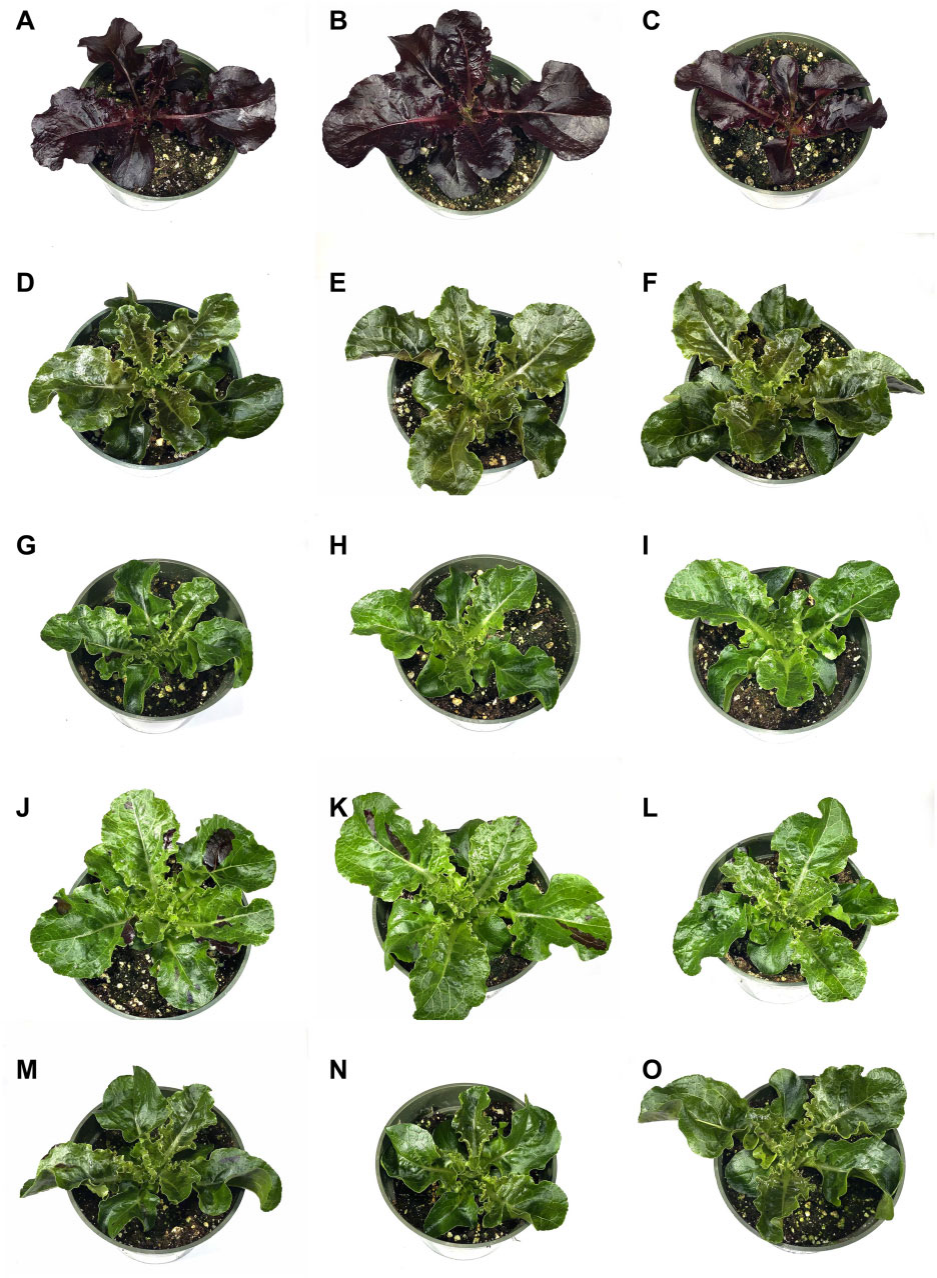
Differences in leaf color between NAR, GSL, GSL-Dark Green (GSL-DG), and GSL-DG with red spots GSL-DG-(r). Images show 35-d old plants kept in a growth chamber equipped with cool fluorescent lights. A–C, NAR. D–F, GSL-DG. G–I, GSL offspring of a GSL-DG-(r) plant. J–L, GSL offspring of a GSL plant. M–O, GSL-DG-(r). Note the presence of irregular red sectors on GSL and GSL-DG-(r) leaves, and their absence on GSL-DG leaves. To capture images, the plants were placed on the same white background and photographed individually using the same camera. During figure assembly, the plants were digitally extracted and placed on a white background.

Related lettuce lines were grouped into four leaf color phenotypes: NAR (uniformly red), GSL (light green with red sectors on leaves), GSL-DG (dark green with no red sectors on leaves), and GSL-DG-(r) (dark green with red sectors on leaves; Figure 1, Supplemental Figure S2).

Segregation for color was systematically determined in self-pollinated offspring of GSL, GSL-DG, and GSL-DG-(r) plants (Supplemental Table S1). Although GSL and GSL-DG plants did not segregate, GSL-DG-(r) offspring could be placed in three phenotypic categories with a Mendelian ratio of approximately 1 GSL: 1 GSL-DG: 2 GSL-DG-(r) (Supplemental Table S1). This indicated that a single gene was responsible for the phenotype, GSL and GSL-DG were homozygous for two different alleles, and GSL-DG-(r) was heterozygous.

### RNA-sequencing indicates that *anthocyanidin synthase* (*ANS*) is differentially expressed in NAR, GSL, and GSL-DG

RNA-sequencing was performed on leaf total RNA from 17-week-old NAR, GSL, and GSL-DG plants grown together in a growth chamber equipped with cool fluorescent lights. This lighting condition was chosen because it promotes flavonoid accumulation (Armas Gutierrez, 2015), and was expected to induce the expression of genes related to flavonoid biosynthesis. Paired end reads of 150 nt were obtained, then trimmed reads were mapped to the lettuce *cv* Salinas V8 reference genome (Reyes-Chin-Wo et al., 2017) and a differential expression analysis was conducted using DESeq2 (Love et al., 2014). The differential expression between NAR and GSL of all the 153 flavonoid biosynthesis related genes identified by Zhang et al. (2017) is shown in Supplemental Table S2, and the differential expression of select genes between the three sequenced lines is shown in Supplemental Table S3. These data revealed that *ANS* expression levels varied drastically between the three sequenced lines. The log2 fold expression change of *ANS* was −7.16 in GSL compared with NAR, −3.25 in GSL-DG compared with NAR, and −3.91 in GSL compared with GSL-DG (Supplemental Table S3). Of the 153 genes associated with flavonoid biosynthesis identified in Zhang et al. (2017), no other genes had a similar expression pattern (i.e. expression levels NAR > GSL-DG > GSL), strongly indicating that the phenotypic differences between red and green lettuce lines were associated with *ANS* expression. The ANS enzyme (also called leucoanthocyanidin dioxygenase, LDOX) converts colorless leucoanthocyanidins to red anthocyanidins (for review, see Saito et al., 2013; Appelhagen et al., 2014), and thus *ANS* was a plausible candidate gene for the mutations in GSL and GSL-DG.

### PCR amplification of *ANS* and its UTRs resulted in identification of transposon insertions in GSL and GSL-DG

Based on the RNA-seq results, primers were designed to amplify *ANS* and its 5′ and 3′ UTRs (Supplemental Table S4). Fragments were amplified from NAR, GSL, and GSL-DG genomic DNA by polymerase chain reaction, and the amplicons were Sanger sequenced. NAR *ANS* and its UTRs (NCBI accession MT674528) were identical to *cv* Salinas *ANS*, apart from the existence of a single point mutation in codon 227. Codon 227 in NAR was GAG, coding for glutamate. In *cv* Salinas, it is TAG, a stop codon that truncates the predicted protein to 226 amino acids compared to the 355 amino acid long NAR protein. However, the NAR *ANS* coding sequence was identical to *ANS* mRNA sequences from three red lettuce lines (NCBI accessions MK522161, AB525912, and MF579549).

**Figure 2:**
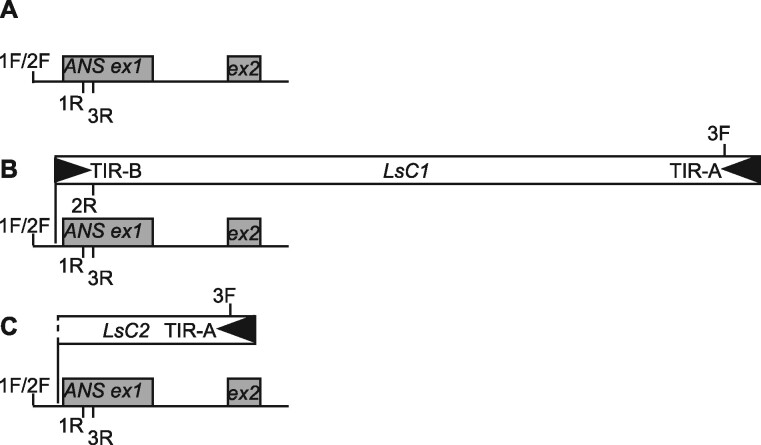
Proportional schematic representation of the *ANS* region in NAR, GSL and GSL-DG, showing transposon insertions in GSL and GSL-DG. A, NAR, no transposon insertion. B, GSL, *LsC1* insertion (3.9 kb) in the *ANS* 5′ untranslated region (5′ UTR). C, GSL-DG, *LsC2* insertion (1.1 kb) in the *ANS* 5′ UTR. *ANS* exon 1 (ex 1) and exon 2 (ex 2) are shown as gray boxes. Transposon TIRs are indicated by filled black triangles. Note that *LsC1* possesses a 5′ and a 3′ TIR, while *LsC2* only has a 3′ TIR. Primer locations are shown for three genotyping primer pairs: 1F/1R (ANS1 F/ANS1 R), 2F/2R (ANS2 F/ANS2 R), and 3F/3R (ANS3 F/ANS3 R). Note that the ANS1 and ANS2 primer combinations share the F primer. For detailed primer location information, see Supplemental Table S5.

Compared with NAR, insertions were found between the 28th and 29th nucleotides upstream of the *ANS* start codon in GSL and GSL-DG, in the *ANS* 5′ UTR. These two nucleotides correspond to linkage group 9 nucleotides 152,766,313 and 152,766,312, respectively, of the lettuce *cv* Salinas V8 genome assembly (NCBI accession CM022526; *ANS* is located on the complementary strand in the genome assembly). Primer walking resulted in the amplification and sequencing of a 3,916-nt insert. A bioinformatics analysis identified the insert sequence as a previously unknown 3,913-nt CACTA (or En/Spm) superfamily transposon with a 5′ACC target site duplication (TSD). We named the transposon *Lactuca sativa CACTA 1* (*LsC1*). *LsC1* harbored 28-nt imperfect TIRs and did not carry any transposase genes, indicating that it is a nonautonomous element. In GSL-DG, an insertion was found at the same site as *LsC1*. This 1,096-nt insertion was composed of 1,094 nt of the 3′ end of *LsC1* (arbitrarily chosen to be the end close to the *ANS* start codon), and the partial TSD CC at the 5′ end of the remaining transposon sequence. No sequence differences were found between this transposon and the homologous region in *LsC1*, and we named this truncated transposon *LsC2*. *LsC2* only had one TIR, the likely cause of its lack of mobility. Figure 2 shows *ANS* and the flanking sequence in NAR (Figure 2A), GSL (Figure 2B), and GSL-DG (Figure 2C). NCBI accession numbers for the *LsC1* and *LsC2* sequences and flanking regions are MT674529 and MT674530, respectively.

### Transposon insertion in the *ANS* 5′ UTR downregulates *ANS* expression and leaf anthocyanin levels

To correlate the presence of *ANS* 5′ UTR transposons to *ANS* expression and leaf flavonoid levels, we grew a population of all available phenotypes (NAR, GSL, GSL-DG(r), GSL-DG) in a shared growth chamber under cool fluorescent lights. Five weeks after sowing, leaf samples were collected from five plants from each line for three different assays detailed below. In addition, the plants were photographed before sampling (Figure 1).

**Figure 3:**
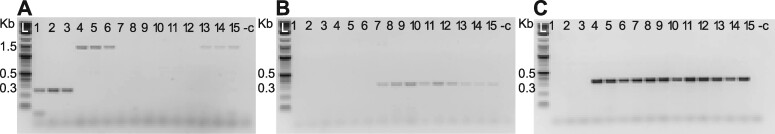
PCR-based genotyping indicates that NAR harbors no transposon, GSL is homozygous for the *LsC1* insertion, GSL-DG is homozygous for the *LsC2* insertion, and GSL-DG-(r) is heterozygous for the *LsC1/LsC2* insertion. Shown are A, ANS1, B, ANS2, and C, ANS3 amplicons in three plants per genotype. 1–3 NAR, 4–6 GSL-DG, 7–9 GSL offspring of a GSL-DG-(r) plant, 10–12 GSL offspring of a GSL plant, 13–15 GSL-DG-(r). Refer to Table 1 for expected fragment sizes. Note faint amplification of the *LsC2*-specific ANS1 amplicon and *LsC1*-specific *ANS2* amplicon in heterozygous samples 13–15, compared to *LsC2* homozygotes 4–6 and *LsC1* homozygotes 7–12.

A PCR-based assay was used to genotype the NAR, GSL, and GSL-DG *ANS* 5′ UTR alleles for the existence of transposon insertion (Table 1, Supplemental Table S5). A schematic representation of primers relative to *ANS* and the transposons is shown in Figure 2. Genotyping confirmed that NAR plants harbored no transposon, GSL plants were homozygous for the *LsC1* insertion, GSL-DG plants were homozygous for the *LsC2* insertion, and GSL-DG-(r) plants were heterozygous for the *LsC1/LsC2* insertion (Figure 3).

**Table 1:** PCR-based genotyping assay to distinguish NAR, GSL, and GSL-DG ANS 5′ UTR. Shown are the three genotyping primer pairs and amplicon size in three genotypes.

Primer Pair	Amplicon Length in NAR (nt)	Amplicon Length in GSL (nt)	Amplicon Length in GSL-DG (nt)
ANS1	280	4,196 (not amplified in assay)	1,376
ANS2	no amplicon	316	no amplicon
ANS3	no amplicon	386	386

RT-qPCR analysis of *ANS* expression was conducted on the abovementioned samples in three technical replicates. *ANS* expression compared to the average of all GSL plants was, on average, 7.65-fold higher in GSL-DG-(r) heterozygotes, 15.56-fold higher in GSL-DG homozygotes, and 155.16-fold higher in NAR plants (Table 2), indicating that a homozygous insertion of *LsC1* in the *ANS* 5′ UTR nearly eliminated the expression of *ANS* and the *LsC2* insertion at the same location allowed for a low level of *ANS* expression.

**Table 2:** Phenotype, genotype, relative ANS expression, and leaf cyanidin content of four lettuce genotypes*.* Average values from five biological replicates ± sd are shown.

Phenotype	ANS Genotype	Relative Expression of *ANS*. Mean of all GSL Plants is 1.0	Cyanidin Concentration (mg cyanidin/g dry leaf)	Relative Cyanidin Concentration. Mean of all GSL Plants is 1.0
GSL (offspring of GSL)	LsC1/LsC1	1.05 ± 0.47	0.69 ± 0.20	1.10
GSL (offspring of GSL-DG-(r)	LsC1/LsC1	0.95 ± 0.20	0.56 ± 0.03	0.90
GSL-DG-(r)	LsC1/LsC2	7.65 ± 0.92	1.10 ± 0.10	1.76
GSL-DG	LsC2/LsC2	15.56 ± 3.12	1.47 ± 0.09	2.34
NAR	+/+	155.16 ± 23.76	19.41 ± 1.00	31.01

**Figure 4:**
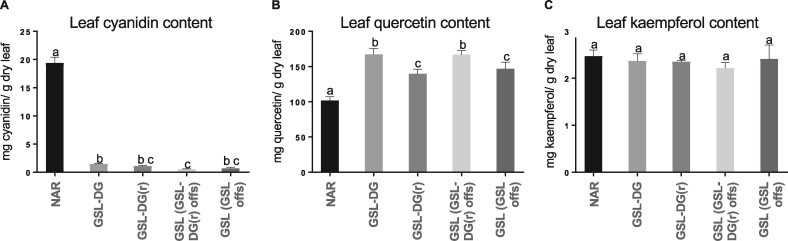
Cyanidin, quercetin, and kaempferol content of NAR, GSL, GSL-DG, and GSL-DG-(r) lettuces. One-way analysis of variance (ANOVA) was performed with Tukey's multiple comparison test; the sample size of each group was *n* = 5. Shown is the mean + sd A, cyanidin, B, quercetin, and C, kaempferol content (mg/g dry leaf) of four lettuce genotypes. Groups are NAR, GSL-DG, GSL-DG-(r), GSL offspring of a GSL-DG-(r) plant, GSL offspring of a GSL plant. Values of *P* < 0.05 were considered significant; values not sharing the same letters are statistically different. For flavonoid concentration values, see Table 2. Note that NAR accumulates higher levels of cyanidin but lower levels of quercetin than GSL and GSL-DG.

**Figure 5:**
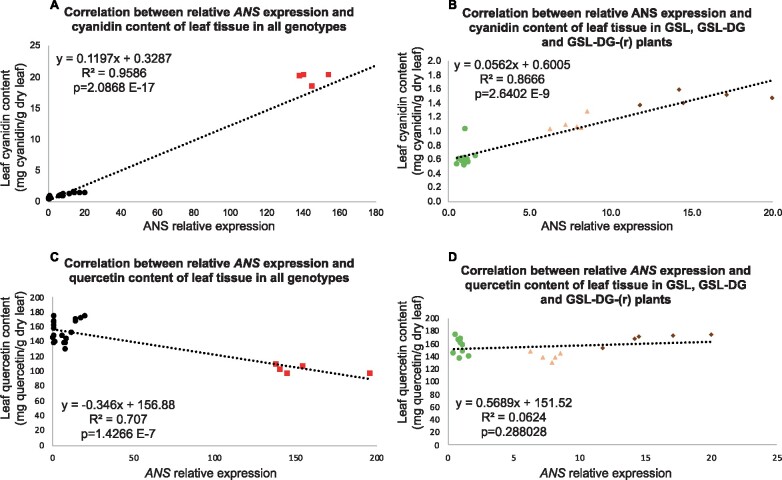
Strong positive correlation between *ANS* expression level and leaf cyanidin content and moderate negative correlation between *ANS* expression level and leaf quercetin content. For all analyses, Pearson correlation coefficient (R) was calculated, *P* < 0.05 was considered significant. *R*^2^ and *P* values are shown for each analysis. A, Correlation between individual plant *ANS* expression levels (relative to GSL average) and leaf cyanidin content (mg cyanidin/g dry leaf) from five NAR, five GSL-DG, five GSL offspring of a GSL-DG-(r) plant, five GSL offspring of a GSL plant, and five GSL-DG-(r) plants. NAR samples are shown as red rectangles, others as black circles. B, Expanded left portion of panel A without NAR data. Samples are shown as: GSL, light green circles; GSL-DG-(r), peach triangles; GSL-DG, brown diamonds. C, Correlation between individual plant *ANS* expression levels (relative to GSL average) and leaf quercetin content (mg quercetin/g dry leaf) in the same plants as shown in panel A. NAR samples are shown as red rectangles, others as black circles. D, Expanded left portion of panel C without NAR data. Samples are shown as: GSL, light green circles; GSL-DG-(r), peach triangles; GSL-DG, brown diamonds. Note the strong positive correlation between *ANS* expression and cyanidin levels for all genotypes. Note the moderate negative correlation between quercetin levels and *ANS* expression when all genotypes are included in the analysis, and lack of correlation if the NAR samples are removed.

Flavonoids were extracted from lyophilized ground leaf samples and the extracts were analyzed using UPLC–MS/MS as described in Gurdon et al. (2019). To precisely quantify flavonoids, the leaf extracts were subjected to acid hydrolysis that removed native glycosylation patterns from flavonoids, reducing them to the aglycones. The major flavonoids of lettuce, namely, cyanidin, quercetin, and kaempferol, were quantified. Cyanidin and quercetin levels (Figure 4, A and B) were negatively correlated (*r* = −0.8489, *P* = 8.15534E-8), whereas there was no significant difference in the kaempferol content among the genotypes (Figure 4C). Compared with the average of all GSL plants, cyanidin levels on average were 1.76-fold higher in GSL-DG-(r) heterozygotes, 2.34-fold in GSL-DG homozygotes, and 31.01-fold higher in NAR plants (Figure 4, Table 2). A strong positive linear correlation between *ANS* expression levels and leaf cyanidin concentration was evident (*r*^2^ = 0.96 for all plants studied, Figure 5A;*r*^2^ = 0.87 when highly expressing NAR samples were removed from the analysis, Figure 5B). A negative linear correlation was also observed between *ANS* expression levels and the accumulation of quercetin in the leaf (*r*^2^ = 0.71 for all plants studied, Figure 5C), but no correlation was evident when NAR samples were removed from the analysis (*r*^2^ = 0.06, Figure 5D). The accumulation of kaempferol showed no correlation with *ANS* expression (*r*^2^ = 0.06, Supplemental Figure S3).

### The lettuce genome harbors more than 1,700 *LsC1* family elements

**Figure 6:**
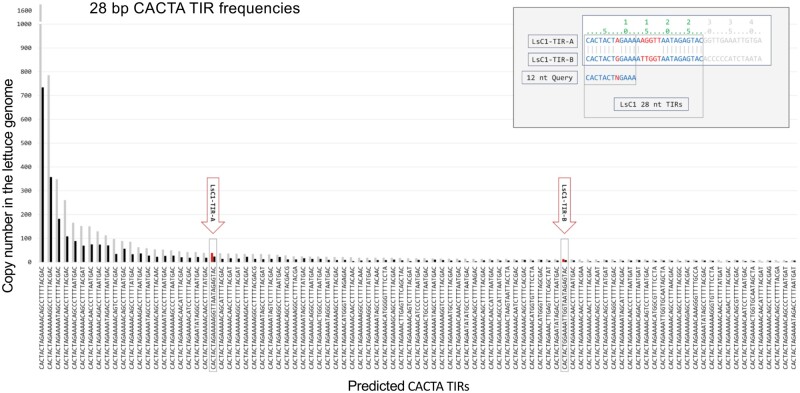
Predicted CACTA-TIR frequencies in the lettuce genome. Figure inset (right upper corner) shows 28-nt-long segments of *LsC1* TIR-A and TIR-B sequences, and the designed 12-nt query used for the BLAST-N search. The main figure displays 28-nt-long TIRs identified in lettuce sorted by their total occurrences in the genome. Gray bars indicate the numbers of all detected TIR candidates, black bars correspond to the TIRs confirmed by forward-reverse orientation and identical TSD tri-nucleotides. Arrows point to the TIR-A and TIR-B sequences specific for *LsC1*. Note that *LsC1* TIRs are different from other most abundant members by their distinct consensus sequence. The majority of lettuce CACTA 28-nt segments harbor a CTTTA/CTTTTA motif, which is not present in *LsC1* TIRs.

Through a combination of homology-based and de novo approaches, 74.2% of the *cv* Salinas reference genome had been shown to consist of repetitive elements (Reyes-Chin-Wo et al., 2017). DNA transposons represented 1.19% of the genome, and elements predicted as belonging to the *En*-*Spm* family (also known as CACTA) transposons comprised 0.47% of the genome (Reyes-Chin-Wo et al., 2017). Although *LsC1* belongs to the CACTA family, previously employed bioinformatics methods (Reyes-Chin-Wo et al., 2017) did not annotate it as a CACTA transposon in the lettuce V8 assembly. A BLAST-N search using the *LsC1* sequence as a query against plant genomes at the NCBI GenBank and CoGe databases revealed the transposon sequence to be unique for the genus *Lactuca* (*L. sativa*, *L. serriola*, *L. saligna*). A BLAST-N homology search with the entire *LsC1* sequence as a query against V8 of the lettuce *cv* Salinas genome assembly resulted in six long high-quality hits that spanned the entire region between the left and right TIRs. All six sequences carried TIRs identical to *LsC1*. The best match (linkage group 7, nucleotides 18,848,559–18,852,487 of the V8 assembly) was 3,929-nt long, 16-nt longer than *LsC1*; furthermore, the two sequences differed in 17 single nucleotide polymorphisms. Intriguingly, this *LsC1* homolog has been recently identified to reside in the first exon of a lettuce *KNOTTED 1* homolog (*LsKN1*), where its presence is linked to up-regulation of *LsKN1* and contributes to the heading phenotype of lettuce (Yu et al. 2020). The next five matches were composed of 3.5 and 0.5-kb *LsC1* segments separated by a 1.9-kb insertion. Three of these five homologs were identical; the fourth only differed from these in one point mutation, while the fifth harbored more point mutations and indels. In addition to these hits, approximately 100 shorter (200 bp–1 kb) segments were identified with up to 80% identity. *LsC1* carried imperfect TIRs of 28 bp (Figure 6, inset), of which we named the 3′ TIR-A and the 5′ TIR-B (chosen arbitrarily, with TIR-A closest to the *ANS* start codon). A BLAST-N search using these conserved 28-nt TIRs as a query identified *Tetu1* (NCBI accession HE604336), an active transposable element isolated from a sunflower (*Helianthus annuus*) mutant (Fambrini et al., 2011, 2014) as the closest characterized active homolog of *LsC1*. However, multiple single nucleotide differences differentiated TIRs of *Tetu1* and *LsC1*.

Sequence analysis indicated that the extreme termini of *LsC1* TIRs (11–12 nucleotides) were common and conserved across a broad set of plant genomes. Therefore, a short segment of 12 nucleotides (Figure 6, inset) was used as a query to identify additional members of this transposon family in the lettuce genome. The uniform length of the conserved TIRs in *LsC1* was 28 nt; therefore, the 12-nt BLAST-N TIR CACTA hits were extended an additional 16 nt into the putative transposon insert. Furthermore, as CACTA inserts have 3-nt TSDs, trinucleotides flanking the transposons were considered putative TSDs. Over 6,700 potential TIRs homologous to *LsC1* TIRs were identified in the lettuce genome. After filtering for (1) proper orientation of TIR pairs; (2) 1–30 kb insert length; and (3) identical TSDs, 1,714 segments were identified as candidate CACTA transposon insertions. All of these candidate CACTA transposons and 28-nt TIRs are listed in Supplemental Table S6. The total length of the characterized 1,714 CACTA elements was 11.6 Mb, the average insertion was 6,800 nt, and the median insertion was 5,600 nt (Supplemental Figure S4). AT-rich TSD trinucleotides were the most abundant within the 64 possible triplet combinations, all of which were detected (Supplemental Figure S5). The set of 1,714 putative transposons had little overlap with previously identified En/Spm segments within the V8 lettuce genome assembly (Reyes-Chin-Wo et al., 2017). Their intersect, found using bedtools (Quinlan, 2014), was only 230 kb for 540 segments and had an average length per segment of 430 nt.

Lettuce CACTA TIR sequences were analyzed for their redundancy and abundance (Figure 6). The most abundant TIR total copy number was 1,635, of which 734 copies were part of a candidate transposon insertion, as defined above. The following five most abundant TIRs had a total copy number of 785, 348, 260, 165, and 151 with 357, 182, 108, 88, and 69 part of a putative transposon, respectively. The most abundant TIR sequences harbored CTTTA/CTTTTA motifs, not present in *LsC1* TIRs. Among the least common putative TIRs, 414 were single copy; 96 had two, 53 had three, 36 had four, and 13 had five copies. The total copy number of *LsC1* TIR-A was 38, of which 23 were part of a putative transposon; TIR-B was even less common with a total copy number of 12, of which only eight were part of a candidate transposon. Of these eight candidate transposons, six were >96% identical to *LsC1*, as described above. These transposons are in rows 849–850, 1785–1786, 2219–2220, 3508–3509, 3671–3672, and 5737–5738 in the Supplemental Table S6 Summary List, and marked there as carrying *LsC1* TIRs.

### The lettuce genome harbors *tnpD* and *tnpA* homologs, allowing active transposition of CACTA elements

Masson et al. (1991) determined that expression of the overlapping alternatively spliced transcripts *tnpD* and *tnpA* is necessary for the transposition of the active maize (*Zea mays*) CACTA transposon *Spm*. *TnpD* and *tnpA* homologs were also identified in active CACTA transposons isolated from other species (for review, see Tian, 2006; Fedoroff, 2013). Therefore, we searched the lettuce genome for *tpnD-tpnA* homologs, using tnpD-tnpA protein sequences from five well-characterized CACTA transposons: one each from soybean (*Glycine max*), snapdragon (*Antirrhinum majus*) and Japanese morning glory (*Ipomoea nil*) and two from maize as queries. A BLAST-P search (expected cutoff 1e-5) detected 73 tnpA and 133 tnpD lettuce candidates. An additional test for the presence of the Pfam domains Transposase_24 and Transposase_21 [http://pfam.xfam.org/family/PF03004, http://pfam.xfam.org/family/PF02992] reduced the set of lettuce candidates to 37 *tnpA* and 108 *tnpD* sequences. RNA expression of these loci was then analyzed in two control *cv* Salinas transcriptomes (BioProject PRJNA12868, Matvienko et al., 2013; BioProject PRJNA553360; Park et al., 2020) and the NAR, GSL, and GSL-DG transcriptome samples of this study (BioProject PRJNA644369). Selected *tnpA* and *tnpD* candidates and results of their RNA expression analysis with CLC Genomics Workbench (QIAGEN) are shown in Supplemental Table S8. At least one pair of loci (LOC111904991 and LOC111904992) had elevated transcription levels of *tnpD* in GSL samples (the *tnpA* transcript from this locus was highly expressed in all samples). Furthermore, contiguous transcription was detected through the *tnpD-tnpA* pair from this locus (Supplemental Figure S6), which is essential for transposase function (Masson et al., 1991).

## Discussion

### GSL and GSL-DG are lettuce *ANS* mutants that accumulate high levels of flavonoids

*ANS* is a single copy gene in lettuce (Zhang et al., 2017). A recent study reported the cloning of four regulatory genes that affect *ANS* expression, and provided a detailed characterization of lettuce lines carrying mutant and wild-type alleles of these regulatory genes (Su et al., 2020). However, to our knowledge, no *ANS* mutants have been characterized in lettuce. GSL and GSL-DG are *ANS* mutants, in which decreased *ANS* expression results in decreased anthocyanin content but elevated levels of quercetin, the major lettuce flavonoid (Figure 4). Lettuce containing high levels of antioxidants, such as flavonoids, may appeal to consumers due to perceived health benefits (Damerum et al., 2015). However, most lettuce consumed is green (Kim et al., 2016), which in general contains much lower flavonoid and antioxidant levels than red cultivars (Llorach et al., 2008). Therefore, GSL, a green romaine line, with high levels of flavonoids and total antioxidants (this study and Armas Gutierrez, 2015) could serve as a nutritionally enhanced substitute for existing romaine lines.

Our analysis also revealed that the green crisphead *cv* Salinas *ANS* harbors an early stop codon compared to NAR and GSL; therefore, *cv* Salinas is an *ANS* nonsense mutant. Intriguingly, while GSL accumulates high levels of flavonoids, *cv* Salinas and other green crisphead cultivars contain low levels (Damerum et al., 2015, Kim et al., 2016) compared with red lettuce lines. These differences are likely due to regulatory factors of flavonoid accumulation.

### Strong linear correlation exists between *ANS* expression and cyanidin accumulation across four isogenic lines

ANS activity is necessary for the formation of red anthocyanidins from colorless leucoanthocyanidins, and the lack of *ANS* expression as well as *ANS* mutations rendering the enzyme nonfunctional result in the lack of anthocyanin accumulation in maize (Menssen et al., 1990), snapdragon (Martin et al., 1991), *Arabidopsis thaliana* (Abrahams et al., 2003; Appelhagen et al., 2011; Bowerman et al., 2012; Appelhagen et al., 2014), pomegranate (*Punica granatum*; Ben-Simhon et al., 2015), raspberry (*Rubus idaeus*; Rafique et al., 2016), mock strawberry (*Duchesnea indica*; Debes et al., 2011), and onion (*Allium cepa*), where three independent mutations impairing ANS function or *ANS* expression have been characterized (Kim et al., 2004; Kim et al., 2005; Kim et al., 2006). However, few reports have quantified the accumulation of anthocyanidins or precursor flavonoids (e.g., quercetin and kaempferol) and their derivatives in *ANS* mutants. Bowerman et al., (2012) found that the transposon-tagged *Arabidopsis thaliana ANS* mutant *tt11-11* accumulated the flavonols kaempferol and quercetin to levels comparable to the wild-type. Similarly, yellow onions harboring an *ANS* mutation likely making the enzyme nonfunctional accumulated similar levels of quercetin to red onions with intact *ANS* (Kim et al., 2005). A white pomegranate line with undetectable *ANS* expression and anthocyanin accumulation had significantly higher levels of kaempferol, quercetin, and catechin in the fruit skin than an anthocyanin-accumulating red line (Ben-Simhon et al., 2015). Although these studies compared one mutant line and a wild-type (Bowerman et al., 2012) or unrelated accessions (Kim et al., 2005; Ben-Simhon et al., 2015), we determined the correlation among four *ANS* genotypes in an isogenic background, enabling us to determine the correlation between *ANS* expression and anthocyanidin and flavonoid accumulation. We found that *ANS* expression was positively correlated with the accumulation of cyanidin in all of the lettuce genotypes included in this study (Figure 5, A and B). Intriguingly, some *ANS* expression and cyanidin accumulation was observed in GSL, despite the homozygous 3.9-kb *LsC1* insertion in the *ANS* 5′ UTR. As the somatic excision frequency of *LsC1* is high, evidenced by ubiquitous small red spots on GSL leaves in addition to larger, irregular red patches (Supplemental Figure S2), we hypothesize that GSL leaf samples collected for RNA expression analysis contained clusters of heterozygous cells with restored *ANS* expression. The three transposon insertion genotypes (*LsC1-ANS/LsC1-ANS*, *LsC1-ANS/LsC2-ANS*, *LsC2-ANS/LsC2-ANS*) and wild-type NAR all showed a distinct *ANS* expression and cyanidin accumulation profile (Figure 5, A and B), indicating that accumulation of the cyanidin in lettuce is directly dependent on *ANS* expression in the four genotypes studied. We did not obtain and thus could not characterize heterozygotes that carried a mutant and a wild-type *ANS* allele.

Similar to our findings, the expression of *chalcone synthase* (*CHS*), the product of which catalyzes the first step in the flavonoid and anthocyanin biosynthesis pathway, was found to be tightly linked to anthocyanin accumulation in the petals of the common morning glory (*Ipomoea purpurea*; Johzuka-Hisatomi et al., 2011). *CHS/CHS*, *CHS/chs*, and *chs/chs I. purpurea* lines all displayed distinct flower coloration phenotypes (Johzuka-Hisatomi et al., 2011) similar to the *ANS* mutant lettuces in this study. However, dose-dependent anthocyanin accumulation or incomplete dominance were not observed in *Ipomoea* lines bearing a mutation in *dihydroflavonol reductase* (*DFR*), a gene that functions downstream of *CHS* and upstream of *ANS*. *DFR/dfr* heterozygotes accumulated *DFR* transcripts and anthocyanins to levels not statistically different from wild-type *DFR/DFR* plants (Johzuka-Hisatomi et al., 2011).

### Green *ANS* mutants accumulate significantly higher levels of quercetin than wild-type red NAR

Accumulation of precursors to significantly higher than wild-type levels has been previously observed in lettuce flavonoid biosynthesis mutants (Gurdon et al., 2019). In the present study, the accumulation of quercetin showed an overall negative correlation with *ANS* expression, and the three transposon insertion genotypes (*LsC1-ANS/LsC1-ANS*, *LsC1-ANS/LsC2-ANS*, *LsC2-ANS/LsC2-ANS*) had significantly higher quercetin levels than the red NAR line. However, there was no correlation between *ANS* expression and quercetin accumulation between transposon insertion genotypes, indicating that other factors must play a role in determining leaf quercetin levels (Figure 5). In addition, kaempferol, a flavonol that accumulates to a much lower level than quercetin in lettuce (Llorach et al., 2008; Gurdon et al., 2019), showed no correlation with *ANS* expression (Supplemental Figure S3).

### *LsC1* is a CACTA-family transposon

Structural and sequence analysis of *LsC1* places it in the CACTA superfamily of transposons. CACTA (also named En/Spm) are DNA transposons exclusive to plants that carry the conserved sequence “CACTA” at both terminal repeat ends and typically have subterminal repeats of 10–20 bp units in direct and inverted orientation (for review, see Bennetzen, 2000, Wicker et al., 2003; Tian, 2006; Wicker et al., 2007). CACTA transposons have been found in many plants and play an important role in genome evolution and stability (Bennetzen, 2000; Tian, 2006). Some CACTA transposons are autonomous, carrying the two open reading frames (ORFs) needed for their excision, the transposase *tnpD*, and the regulatory protein *TnpA*, which binds to the subterminal repeats (Fedoroff, 2013). However, most CACTA transposons do not carry either ORF and are thus nonautonomous (Wicker et al., 2003; Kawasaki and Nitasaka, 2004; Tian, 2006; Hoshino et al., 2016; Catoni et al., 2019). CACTA transposons frequently capture fragments of the host genome and related CACTA transposons may carry identical subterminal regions with unrelated sequences in between (Kawasaki and Nitasaka, 2004; Tian, 2006; Catoni et al., 2019). For example, in *I. nil* 28 transposon types were identified in the CACTA transposon family *Tpn1* based on restriction mapping: the types shared subterminal regions and captured a wide range of endogenous gene fragments (Kawasaki and Nitasaka, 2004).

Reyes-Chin-Wo et al. (2017) identified more than 24,000 putative CACTA (En/Spm) segments totaling 12 Mb in the *cv* Salinas lettuce genome. However, these candidates were not subject to validation or any further detailed analysis, nor were active CACTA transposons detected and characterized in *cv* Salinas or any other lettuce variety. *Tetu1*, a transposable element isolated from sunflower (Fambrini et al., 2011; Fambrini et al., 2014), was found to be the closest active transposon homolog of *LsC1*, based on BLAST-N results using the 28-nt conserved TIRs of *LSC1* as the query. However, multiple *Tetu1* and *LsC1* TIRs differed in multiple nucleotides, and apart from the terminal regions, *Tetu1* and *LsC1* were not homologous.

### *LsC2* is an inactive derivative of *LsC1* that was isolated immediately after its formation

Active transposition of CACTA elements has been described from only a handful of species such as maize (Paz-Ares et al., 1986; Menssen et al., 1990), soybean (Zabala and Vodkin, 2008; Xu et al., 2010), petunia (*Petunia hybrida*, Snowden and Napoli, 1998), four o'clock (*Mirabilis jalapa*, Suzuki et al., 2014), snapdragon (Luo et al., 1991), Japanese morning glory (for review, see Hoshino et al., 2001; Iida et al., 2004), and sunflower (Fambrini et al., 2011; Fambrini et al., 2014). In the abovementioned reports, somatic excision of transposons created leaf sectors with a phenotype different from surrounding tissue, or germinal excision led to the appearance of wild-type revertants. These events allowed the linkage of the wild-type phenotype to the excision of the transposon hypothesized to be responsible for the mutant phenotype and identifying an active CACTA element in lettuce. This identification was facilitated by the easily detectable phenotype of red spots on an otherwise green leaf (Figure 1, Supplemental Figure S1), resulting from somatic excision of *LsC1* from the *ANS* 5′ UTR and restored *ANS* expression.

Inactive CACTA elements do not result in a dynamic mutable phenotype, necessitating methods of discovery different from active element detection. Inactive elements have been identified from stable mutants (e.g. Nitasaka, 2003; Kim et al., 2015) by comparing a candidate gene sequence from wild-type and mutant lines, and discovering a transposon insertion in a candidate gene. Furthermore, uncommon autonomous elements may be identified bioinformatically by searching for homologs of *tnpA* and *tnpD* (Hoshino et al., 2016). However, the overwhelming majority of CACTA elements lack these ORFs and are nonautonomous (Kawasaki and Nitasaka, 2004; Hoshino et al., 2016). Such elements are typically identified by searching for repeat sequence using the TIR regions as queries because the “middle” regions of transposons carry noncoding genome fragments from the host species (Wicker et al., 2003; Kawasaki and Nitasaka, 2004; Hoshino et al., 2016). For example, Hoshino et al. (2016) identified 399 *Tpn1* homologs in the *I. nil* genome by querying the assembled genome sequence with a 28-nt TIR sequence, identifying “nearby” TIRs in the genome in the expected orientation that had the same TSDs and nominating them as transposons. The shortest identified element was 161 bp and the longest was 40,619 bp (Hoshino et al., 2016). However, analyses, such as those performed by Hoshino et al., (2016) will miss partial CACTA elements with just one TIR, such as *LsC2* reported here. *LsC2* is a derivative of the intact *LsC1* that lacks 2,820 nt from the 5′ end of *LsC1*, including the 5′ TIR. Therefore, detecting this element using bioinformatics would be challenging. However, transposition of *LsC1* to the *ANS* 5′ UTR, as well the formation of *LsC2* by incomplete *LsC1* excision occurred in our greenhouses, resulting in lines that (likely) differed only in one locus. The existence of isogenic lines only differing in their transposon insertion locus allowed facile identification of the insertion sites.

Though we have no definitive proof for the activation of *LsC1* in GSL, we hypothesize that the tissue culture methods used to develop its parent line, NAR (Cheng et al., 2014), activated the transposon, and serendipitous insertion upstream of *ANS* created a visible phenotype. Tissue culture has been shown to activate silent transposons, such as in *Saintpaulia* (Sato et al., 2011), maize (Brettell and Dennis, 1991), and barley (*Hordeum vulgare*; Orlowska et al., 2016), likely linked to changed methylation patterns.

### The *LsC1* family contains more than 1,700 putative members in lettuce

*LsC1* was not found within previously annotated transposons at the time of publication of the V8 genome (Reyes-Chin-Wo et al, 2017). Using the *LsC1* transposon as a query against the *cv* Salinas lettuce reference genome assembly (GCF_002870075.1), yielded one nearly identical putative transposon. Furthermore, five longer putative transposons shared over 96% identity with *LsC1*. Therefore, we speculated that these six sequences were homologs of *LsC1*. Intriguingly, the *cv* Salinas homolog with the highest sequence identity to *LsC1* is inserted in the first exon of a *KNOTTED 1* homolog (*LsKN1*). Recent analysis indicates that insertion of the element during domestication of lettuce has resulted in upregulation of *LsKN1* and contributed to the heading phenotype, including in *cv* Salinas (Yu et al. 2020). Upregulation of *KN1* by the *LsC1* homolog insertion contrasts with how *LsC1* represses the expression of *ANS* in GSL. We hypothesize that this contrasting effect on gene expression may depend on the location of the insertion: the 5′ UTR of *ANS* in GSL, and an exon of *KN1* in *cv* Salinas. Interestingly, the *cv* Salinas *LsC1* homolog was not reported to be active (Yu et al. 2020).

Using the 28-nt *LsC1* TIRs to search the lettuce genome assembly, we identified 1,714 sequences between correctly oriented TIRs and TSDs. It is possible that at least some of the TIRs without complementary reverse counterparts are in fact ends of derivative truncated transposons similar to *LsC2*. As the “middle” regions of nonautonomous CACTA elements typically carry gene fragments from the host species (Wicker et al., 2003; Kawasaki and Nitasaka, 2004; Hoshino et al., 2016), determining the exact boundaries of such “broken” derivative elements is challenging without high quality, long read-based genomes from multiple lettuce accessions.

The CACTA transposon insertions identified by us have only a small overlap (2%) with previously annotated En/Spm segments in the lettuce genome assembly (Reyes-Chin-Wo et al, 2017). In that study, transposon candidates were identified using a simple homology search with RepeatMasker, while we employed a multifactor approach considering TIR orientation, TSD presence, and insert length. This approach allowed for the discovery and characterization of full length putative CACTA inserts, rather than just short segments homologous to query sequences.

### Putative genes required for transposon excision are found in lettuce and expressed in GSL

Detection of *tnpD* and *tnpA* in the transcriptome is a bioinformatics challenge because *tnpD* usually has near zero expression levels, whereas *tnpA* has a complex multiexon structure. Furthermore, *tnpA* is highly diverged between various species (Zabala and Vodkin, 2008) and the success of a homology search depends on query sequence selection and search parameters. Although we queried the lettuce genome with *tnpD* and *tnpA* genes carried by well-characterized active CACTA transposons from multiple species, our results are limited by the number of such elements identified so far. Nevertheless, the presence of Pfam domains and observed expression profiles indicate that the lettuce *tnpD-tnpA* pair LOC111904991 and LOC111904992 may act as an active transposase complex (Supplementary Table S8). According to Masson et al. (1991), contiguous transcripts through the *tnpD-tnpA* pair are required for a functionally active transposase complex, and such contiguous transcripts were detected from LOC111904991 and LOC111904992 in GSL RNA-seq samples. Therefore, although we lack direct evidence, these transcripts are candidates to encode the active transposase complex responsible for *LsC1* transposition.

In a study of another Asteraceae species, sunflower, bioinformatic search for *Tetu1*-like CACTA elements resulted in the identification of 707 putative transposons, of which 84 were complete with two TIRs (Ventimiglia et al., 2020). The elements carried a total of 39 transposase sequences (*TnpA* and *TnpD* homologs were not distinguished). Of these 39 putative transposases, only 10 were actively transcribed, but expression levels were very low apart from one locus (Ventimiglia et al., 2020).

### Summary and conclusions

We report the identification and characterization of active transposon *LsC1* and its truncated immobilized derivative *LsC2* from lettuce *ANS* mutants accumulating high levels of flavonoids. Detailed bioinformatics analysis has demonstrated that these elements belong to a transposon family not previously characterized in the lettuce genome. A complex integrated approach to mine the genome for complete CACTA insertions identified more than 1,700 putative transposons belonging to the *LsC1* family. Further work is required to study the dynamics of these mobile elements within lettuce varieties to understand the evolution of repetitive elements in the lettuce genome and to conduct reverse genetic analyses in this important crop. In addition, the *ANS* mutants reported here accumulate high levels of flavonoids and could serve as a nutritionally enhanced substitute of existing romaine lines.

## Materials and methods

### Plant lines

NAR was developed from lettuce (*Lactuca sativa*) *cv* Annapolis using tissue culture selection (Cheng et al., 2014), and was thereafter maintained by selfing. The first GSL individual was found as a green seedling among red NAR offspring (Armas Gutierrez, 2015), and the line was thereafter maintained by selfing. The first GSL-DG(r) plant was found as an olive-green seedling among GSL offspring. This plant segregated to GSL-DG-(r), GSL-DG, and GSL plants, and these lines were maintained by selfing. GSL-DG-(r) offspring segregated, whereas GSL and GSL-DG were stable phenotypes (Supplemental Figure S1).

### Growth conditions for line maintenance

Seeds were sown in Sun Gro Propagation Mix (Sun Gro Horticulture, Agawam, MA, USA) in plastic trays, and these trays were placed in growth chambers equipped with cool fluorescent lights, as described in Gurdon et al. (2019). Plants were transplanted to 10 cm diameter pots (Nursery Supplies, USA) filled with Sun Gro Professional Growing Mix (Sun Gro Horticulture, Agawam, MA, USA) 3–6 weeks after sowing. Twelve to eighteen weeks after sowing, the plants were transplanted again to 23-cm diameter pots (Nursery Supplies, USA) filled with Sun Gro Professional Growing Mix (Sun Gro Horticulture, Agawam, MA, USA), moved to the research greenhouse at Rutgers New Jersey Agricultural Experiment Station (NJAES), and grown to seed as described in Gurdon et al. (2019). Individual inflorescences were collected and dried, then the seeds were threshed and stored at 4°C in the dark.

### Growth conditions for RNA-seq analysis

Seeds of NAR, GSL, and GSL-DG were sown in Sun Gro Propagation Mix (Sun Gro Horticulture, Agawam, MA, USA) in plastic trays, and these trays were placed in growth chambers equipped with cool fluorescent lights, as described in Gurdon et al. (2019). Plants were transplanted to 10 cm diameter pots filled with Sun Gro Professional Growing Mix (Sun Gro Horticulture, Agawam, MA, USA) 34 d after sowing, then transplanted again using the same growing mix into 23 cm diameter pots (Nursery Supplies, USA) 51 d after sowing. Leaf samples for RNA-seq analysis were collected in triplicate from one of the three youngest leaves (depending on leaf size) of three GSL, three NAR, and three GSL-DG plants 121 d after sowing.

### Growth conditions for genotyping, phytochemical, and RNA expression analysis

Seeds of NAR, GSL, GSL-DG, and two GSL-DG-(r) lines were sown in Sun Gro Propagation Mix (Sun Gro Horticulture, Agawam, MA, USA) in plastic trays, and these trays were placed in growth chambers equipped with cool fluorescent lights, as described in Gurdon et al. (2019). Under these growth conditions, NAR develops a deep red color, GSL a light green color, and GSL-DG an olive color. Plants were transplanted to 10-cm diameter pots filled with Sun Gro Professional Growing Mix 19 d after sowing (Sun Gro Horticulture, Agawam, MA, USA). The segregating GSL-DG-(r) offspring were grouped as GSL, GSL-DG-(r), or GSL-DG. Thirty-five days after sowing, the plants were photographed. On the same day, tissue samples for phytochemical analysis were collected from five NAR individuals, five GSL individuals, five GSL-DG individuals, five individuals that were GSL offspring of a GSL-DG-(r) individual, five individuals that were GSL-DG offspring of a GSL-DG-(r) individual, and five individuals that were GSL-DG-(r) offspring of a GSL-DG-(r) individual. For the phytochemical analysis, the third–to fifth or third–sixth (if leaves were small) youngest leaves were harvested from each plant and pooled. Thirty-seven days after sowing, three samples were harvested from the youngest visible leaf (or if the youngest leaf was very small, the second youngest leaf) from each plant for DNA and RNA isolation. For GSL plants, large red patches on the leaves were avoided.

### Acid hydrolysis, UPLC–MS/MS analysis, and quantification of flavonoid aglycones from leaf tissue

After their fresh weight was recorded, leaf samples were stored at −80°C, then lyophilized. After lyophilization, the dry weight of the samples was recorded, and the samples were ground to a fine powder using a mortar and pestle. Acid hydrolyzed extracts from 50-mg lyophilized leaf powder per plant were prepared according to the method described by Hertog et al. (1992), with modifications as described in Gurdon et al. (2019). UPLC–MS/MS was conducted on the extracts, and flavonoid aglycones were quantified according to Gurdon et al. (2019).

### RNA-sequencing and analysis

Leaf samples were collected from one of the three youngest leaves of three GSL, three NAR, and three GSL-DG plants, and stored at −80°C. Total RNA was isolated using a QIAGEN Plant RNA Kit (QIAGEN, Hilden, Germany) according to the manufacturer's instructions. RNA-sequencing and subsequent sequence processing was performed by Genewiz (South Plainfield, NJ), where 150 bp paired-end reads were obtained using an Illumina Hi-Seq (Illumina, San Diego, CA) sequencer. Raw reads were deposited in the NCBI Sequence Read Archive database (BioProject Accession PRJNA644369). The number of raw reads ranged from 133,281,223–165,971,010 per sample, equaling 39,984–49.791 Mb. Sequence reads were trimmed to remove adapter sequences and low-quality nucleotides with Trimmomatic v.0.36 (Bolger et al., 2014). Trimmed reads were mapped to V8 of the lettuce *cv* Salinas reference genome (Reyes-Chin-Wo et al., 2017) using STAR aligner (Dobin et al., 2013) v.2.5.2b. Unique gene hit counts were calculated by using featureCounts (Liao et al., 2014) from Subread package (Liao et al., 2013) v.1.5.2. Only unique reads that fell within exon regions were counted. After extraction of gene hit counts, the gene hit counts table was used for downstream differential expression analysis. Using DESeq2 (Love et al., 2014), expression of genes was compared between NAR, GSL, and GSL-DG. A Wald test was used to generate *P*-values and log2 fold changes. Genes with an adjusted *P*-value < 0.05 and absolute log2 fold change > 1 were called as differentially expressed. For Supplemental Tables S2 and S3, the flavonoid biosynthesis pathway genes identified by Zhang et al. (2017) were extracted from the chart of differentially expressed genes.

### Sequencing the *ANS* region in NAR, GSL, and GSL-DG

Total cellular DNA was isolated from leaves of NAR, GSL, and GSL-DG using a modified cetyltrimethylammonium bromide (CTAB) method (Murray and Thompson, 1980). Total DNA was used as a template to amplify the entire coding sequence of *ANS* and its up-and downstream regions using the following primer combinations: ANS_4341F/ANS_5358R, ANS_5176F/ANS_6508R, ANS_6318F/ANS_7550R, ANS_7341F/ANS_8749R, ANS_8419F/ANS_9690R, and ANS_9358F/ANS_10968R (Supplemental Table S4). The following PCR program was used for amplification: 5 min at 94°C; 34 cycles of 30 s at 94°C, 30 s at 60°C, 120 s at 72°C; and a final extension at 72°C for 10 min. PCR products were either isolated from gels using a ZymoClean Gel isolation kit (Zymo Research, Irvine, CA) according to the manufacturer's instructions or treated with ExoSAP-IT (Affymetrix) according to the manufacturer's instructions. Purified fragments were Sanger sequenced at GENEWIZ (South Plainfield, USA), and raw sequences were assembled using SeqMan Pro (DNASTAR, Madison, WI). The transposon insertion in GSL was Sanger sequenced at GENEWIZ (South Plainfield, USA) in two primer walking steps from ANS_8419F/ANS_9690R amplicons, using the primers ANS_insert_669F, ANS_insert_1469F, ANS_insert_1939R, and ANS_insert_937R (Supplemental Table S4).

### Genotyping NAR, GSL, GSL-DG, and GSL-DG(r) lines

Total cellular DNA was isolated from five NAR plants, five GSL plants, five GSL-DG plants, five plants that were GSL offspring of a GSL-DG-(r) plant, five plants that were GSL-DG offspring of a GSL-DG-(r) plant, and five plants that were GSL-DG-(r) offspring of a GSL-DG-(r) plant, using a modified CTAB method (Murray and Thompson, 1980). Genotyping was conducted using three primer combinations (Supplemental Table S5), and the following PCR program for amplification: 5 min at 94°C; 28 cycles of 15 s at 94°C, 18 s at 56°C, and 60 s at 72°C; and a final extension at 72°C for 5 min. The amplicons were run next to 1 Kb Plus DNA Ladder (Thermo Fisher, Waltham, MA) on 1.5% agarose (wt/vol) Tris-acetate-EDTA gels (Green and Sambrook, 2012).

### Quantification of *ANS* expression by RT-qPCR

Total cellular RNA was isolated from the same plants as used for genotyping with the RNeasy Plant Mini Kit Kit (QIAGEN, Hilden, Germany) according to the manufacturer's instructions. Total RNA was quantified using a NanoDrop UV–Vis spectrophotometer (Thermo Fisher Scientific, Waltham, MA), and used as a template for cDNA synthesis using the High-Capacity cDNA Reverse Transcription Kit (Thermo Fisher Scientific, Waltham, MA). cDNA synthesis was performed according to the manufacturer's instructions, with 1-μg RNA in 1 μL reaction mix. RT-qPCR was performed using the Power SYBR Green Master Mix (Thermo Fisher Scientific, Waltham, MA), according to the manufacturers' instructions, with 1-μL 10× diluted cDNA in a 10-μL reaction mix. RT-qPCR primers are listed in Supplemental Table S7; the internal control *Tip41* was identified by Borowski et al. (2014) as a stable reference gene in lettuce for RT-qPCR applications, and Tip41_1 primer sequences were taken from Borowski et al. (2014). The primer pair ANS-q1 was designed anew. RT-qPCR was done in triplicate for every biological sample and primer pair on a QuantStudio 3 system (Thermo Fisher Scientific, Waltham, MA). Results from multiple plates were uploaded to the Thermo Fisher Connect cloud (www.thermofisher.com/us/en/home/digital-science/thermo-fisher-connect.html) and analyzed together. One of the GSL samples was arbitrarily selected as a reference sample, and GSL was selected as a reference biogroup. The confidence level was 95.0, the Benjamini–Hochberg false discovery rate for *P*-values was one, and the default auto threshold and baseline setting was used for the analysis. A Pearson correlation analysis between flavonoid levels and *ANS* expression was conducted in Microsoft Excel (Microsoft, Redmond, WA, USA).

### Identification of CACTA elements in lettuce by homology search for TIRs

Putative CACTA transposon TIRs were identified by homology search using the 12-nt sequence CACTACTNGAAA as a query in a BLAST-N (Altschul et al., 1990) search versus lettuce genome reference V7/V8 GCF_002870075.1 (BLAST version 2.2.26 with parameters: blastall -p blastn -V T -F F -W 7 -e 1000 -z 10000 -b 24000 -v 24000). Position 8 of the query distinguishes TIR-A and TIR-B; thus, an N was used to allow for searching with a single query. The BLAST-N output was processed with tcl_blast_parser (https://github.com/alex-kozik/atgc-tools/blob/wiki/tcl_blast_parser.md) and then TIR candidates were extracted by extending BLAST-N alignments to a uniform 28-nt length with a custom script (https://github.com/alex-kozik/atgc-01/tree/main/CACTA). TIR segments along with adjacent trinucleotides were compiled in a table, imported into Microsoft Excel (Microsoft, Redmond, WA, USA), and queried using Excel internal features (Supplemental Table S6). Then sequences were nominated as putative transposons if they were (1) in the same contig, (2) located between TIR pairs in inverted orientations, (3) adjacent identical TSD trinucleotides were present, and (4) the sequence length was within the expected size range of 1,000–30,000 nt. Genome coordinates were then compiled into GFF and BED files. Overlap for genome features was analyzed with bedtools (Quinlan 2014) using the intersect function.

### Identification of putative *TnpA* and *TnpD* homologs in the lettuce genome

Ten tnpD–tnpA protein sequences of very well-characterized CACTA elements (two tnpD-tnpA pairs in maize [*Zea mays*, GenBank accessions AAA66266.1 and AAA66268.1, Pereira et al. (1986); and AAG17043.1 and AAG17044.1, Bercury et al. (2001)], snapdragon [*Antirrhinum majus*, CAA40555.1 and CAA40554.1, Nacken et al. (1991)], soybean [Glycine max, EU190440, Zabala and Vodkin (2008)], and Japanese morning glory [Ipomoea nil, BAV56702.1 and BAV56701.1, Hoshino et al. (2016)] were used as BLAST-P (Altschul et al., 1990) queries to search predicted genes of complete lettuce genome [NCBI GenBank RefSeq V7 https://www.ncbi.nlm.nih.gov/genome/annotation_euk/Lactuca_sativa/100/], with an expectation cutoff of 1e-5. An additional test for the presence of Pfam domains Transposase_24 (tnpA) and Transposase_21 (tnpD) [http://pfam.xfam.org/family/PF03004 and http://pfam.xfam.org/family/PF02992] was used to select putative transposases carrying these motifs among those identified in the BLAST-P search.

### Accession numbers

The following nucleotide sequences have been deposited in NCBI: NAR *ANS* including 5′ UTR (MT674528), GSL NAR *ANS* including 5′ UTR *LsC1* insertion (MT674529), and GSL-DG partial NAR *ANS* including 5′ UTR *LsC2* insertion (MT674530). RNA-sequencing raw reads from three biological replicates per genotype were deposited in the NCBI Sequence Read Archive database as BioProject Accession PRJNA644369 (NAR: SAMN15458146, SAMN15458147, SAMN15458148, GSL-DG: SAMN15458149, SAMN15458150, SAMN15458151, GSL: SAMN15458152, SAMN15458153, SAMN15458154). The previously published reference genome assembly used in this study is available at NCBI GenBank under the following accessions: GCF_002870075.1 (RefSeq V7 scaffolds) and GCA_002870075.2 (V8 whole chromosome level).

## Supplemental data

The following supplemental materials are available in the online version of this article.

**Supplemental Table S1.** Segregation of leaf color indicates GSL-DG-(r) plants are heterozygous, while GSL and GSL-DG are homozygous for the gene responsible for the phenotype.

**Supplemental Table S2.** Differential expression of 153 flavonoid biosynthesis associated genes identified in Zhang et al. (2017) between NAR and GSL.

**Supplemental Table S3.** Differential expression of select flavonoid biosynthesis associated genes identified in Zhang et al. (2017).

**Supplemental Table S4.** Primer sequences used to amplify *ANS*, its *UTRs*, and the *LsC1* insertion.

**Supplemental Table S5.** Genotyping assay to distinguish NAR, GSL, and GSL-DG *ANS* 5′ UTR.

**Supplemental Table S6.** Predicted CACTA transposons in the *cv* Salinas genome (GenBank RefSeq GCF_002870075.1).

**Supplemental Table S7.** RT-qPCR primers used to amplify *ANS* and the internal control *TIP41.*

**Supplemental Table S8.** Putative *tnpA* and *tnpD* homologs.

**Supplemental Figure S1.** Pedigree of lettuce anthocyanin biosynthesis mutants GSL and GSL-DG.

**Supplemental Figure S2.** Red spots are ubiquitous on GSL leaves, indicating frequent somatic excision of *LsC1*.

**Supplemental Figure S3.** No correlation exists between *ANS* expression level and leaf kaempferol content.

**Supplemental Figure S4.** Insert length distribution of 1,714 putative *LsC1* family transposons in the *cv* Salinas lettuce genome.

**Supplemental Figure S5.** Target site duplication (TSD) frequencies of putative lettuce CACTA transposons in the *cv* Salinas lettuce genome.

**Supplemental Figure S6.** Expression from a putative *tnpD-tnpA* locus in *cv* Salinas, NAR, GSL, and GSL-DG shows contiguous transcription across the putative genes in two of three GSL samples.

## Supplementary Material

kiab143_Supplementary_Data

## Notes

C.G. and I.R. conceived the research plans; C.G. isolated the GSL-DG plant, designed, and performed all bench research apart from HPLC–MS experiments, analyzed data, and wrote most of the manuscript with contributions from all authors; A.P. performed all HPLC–MS experiments and analysis; I.A. isolated the original GSL plant and performed preliminary phytochemical analyses; A.K. conducted all bioinformatics analyses and wrote the part of the manuscript reporting these results. R.T. conducted preliminary bioinformatics analysis. R.W.M. supervised R.T. and A.K. and assisted with writing the manuscript. I.R. supervised the project and assisted with writing the manuscript. C.G. agrees to serve as the author responsible for contact and ensures communication.

The author responsible for distribution of materials integral to the findings presented in this article in accordance with the policy described in the Instructions for Author (https://academic.oup.com/plphys/pages/general-instructions) is: Csanad Gurdon (gurdon@sebs.rutgers.edu).
