# Supplementary material for: Isolating an active and inactive CACTA transposon from lettuce color mutants and characterizing their family
Source: Plant Physiol. 2021 Mar 26;186(2):929–44. doi: 10.1093/plphys/kiab143 (PMC8195511; doi:10.1093/plphys/kiab143)
Supplement: kiab143_Supplementary_Data [file kiab143_supplementary_data.zip › pp.01736.2020-s01.pdf]

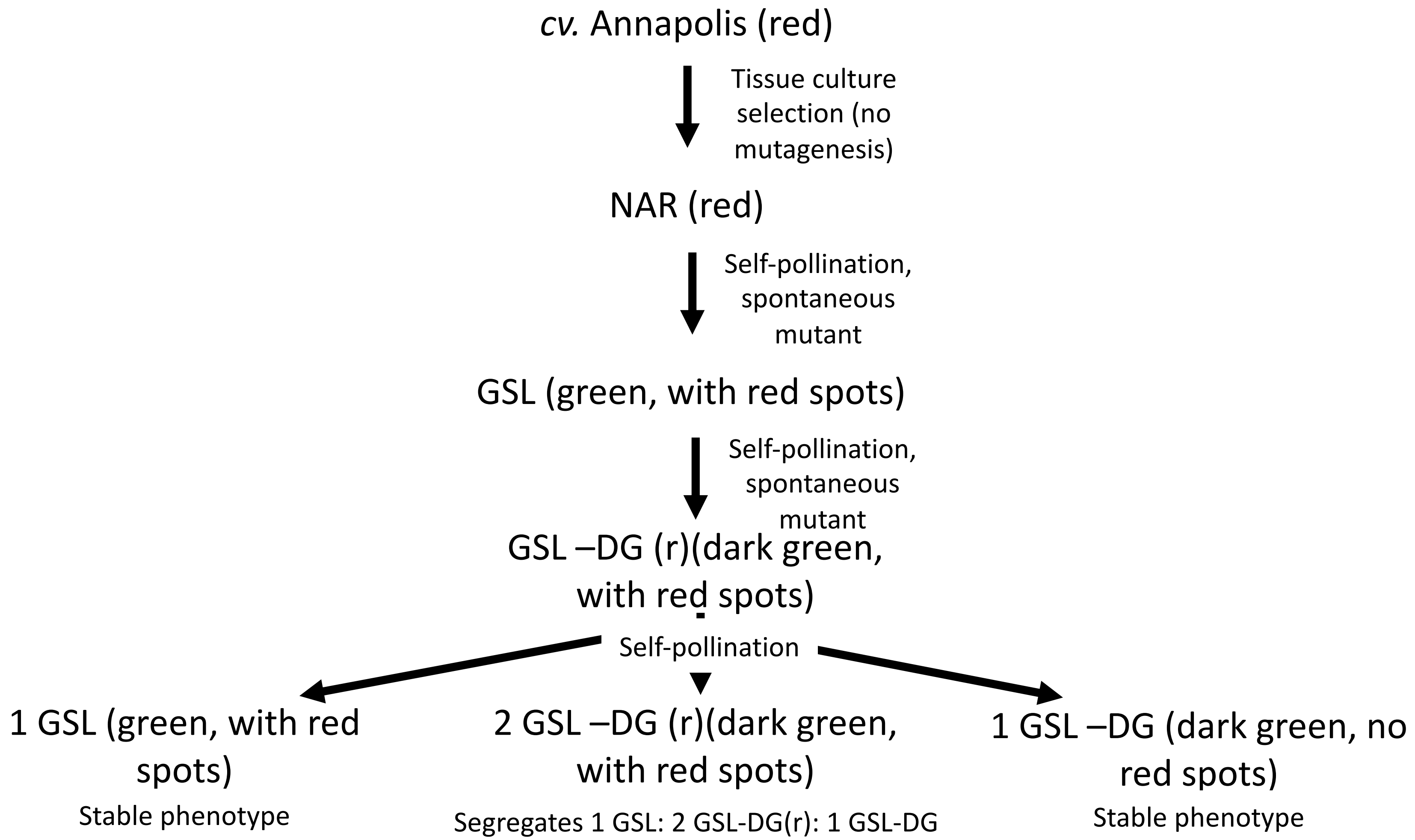

Supplemental Figure S1. Pedigree of lettuce anthocyanin biosynthesis mutants GSL and GSL-DG.

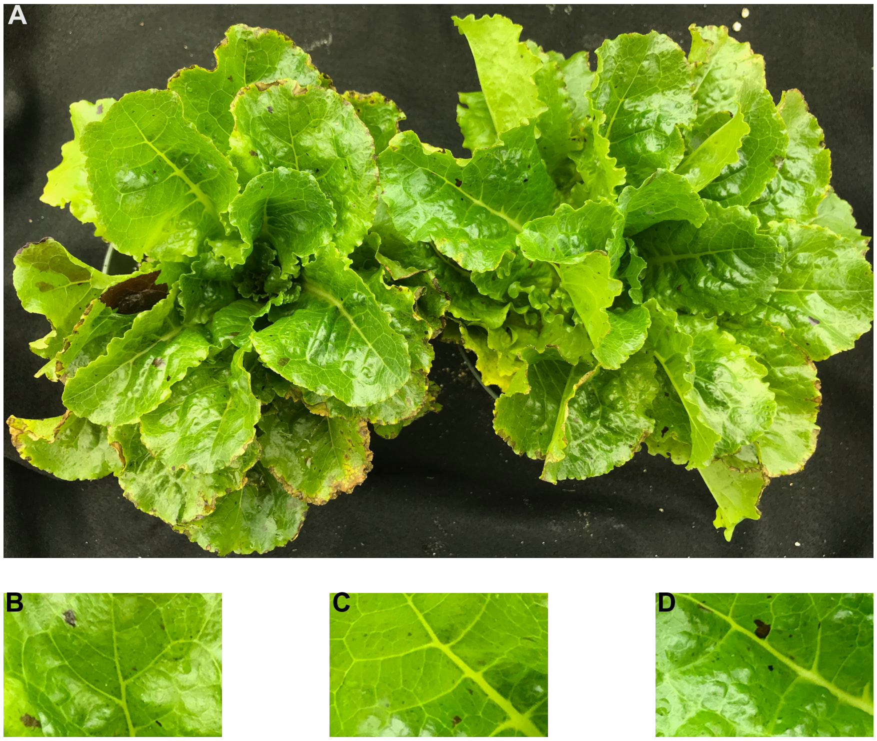

Supplemental Figure S2. Red spots are ubiquitous on GSL leaves, indicating frequent somatic excision of *LsC1*. Shown are A, two grow chamber grown GSL plants 115 days after sowing. B, C, D, Close-up details from panel A.

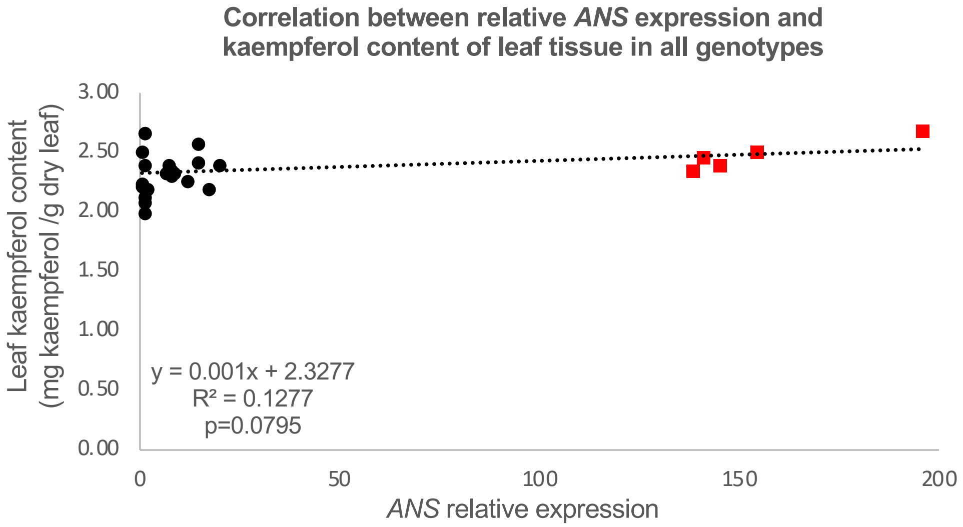

Supplemental Figure S3. No correlation exists between *ANS* expression level and leaf kaempferol content. Shown is the lack of correlation (Pearson correlation, p = 0.0795) between individual plant *ANS* expression levels (relative to GSL average) and leaf kaempferol content (mg kaempferol / g dry leaf) from 5 NAR, 5 GSL-DG, 5 GSL offspring of a GSL-DG-(r) plant, 5 GSL offspring of a GSL plant, and 5 GSL-DG-(r) plants. NAR samples are shown as red rectangles, others as black circles.

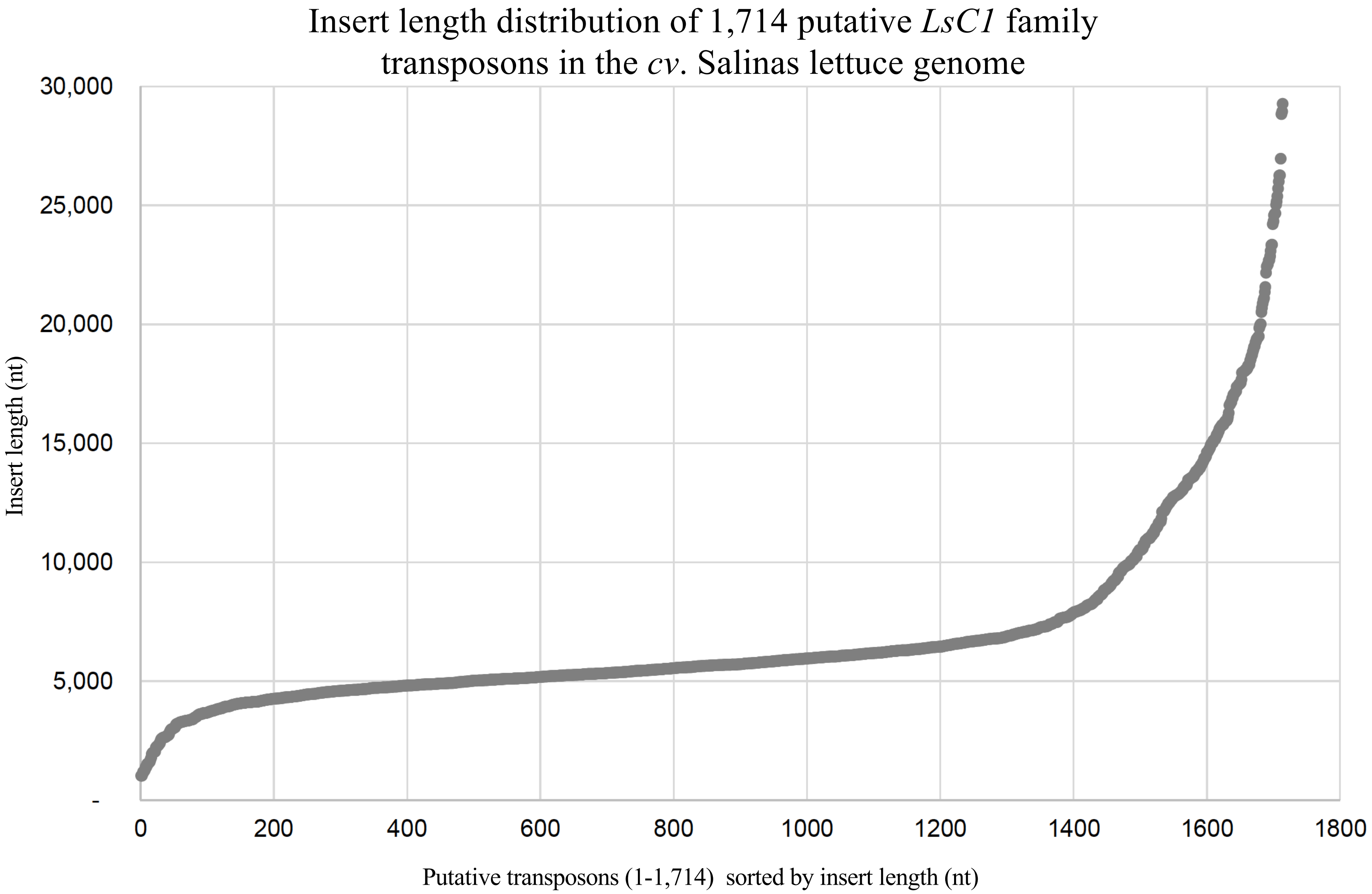

Supplemental Figure S4. Insert length distribution of 1,714 putative LsC1 family transposons in the *cv*. Salinas lettuce genome (GenBank RefSeq GCF_002870075.1). The transposons were confirmed by flanking TIRs and identical TSD tri-nucleotides. Putative transposon insertions are sorted by their length on the X-axis; the insert length values are displayed on the Y-axis.

TSD frequencies of putative *LsC1* family transposons in the *cv*. Salinas lettuce genome

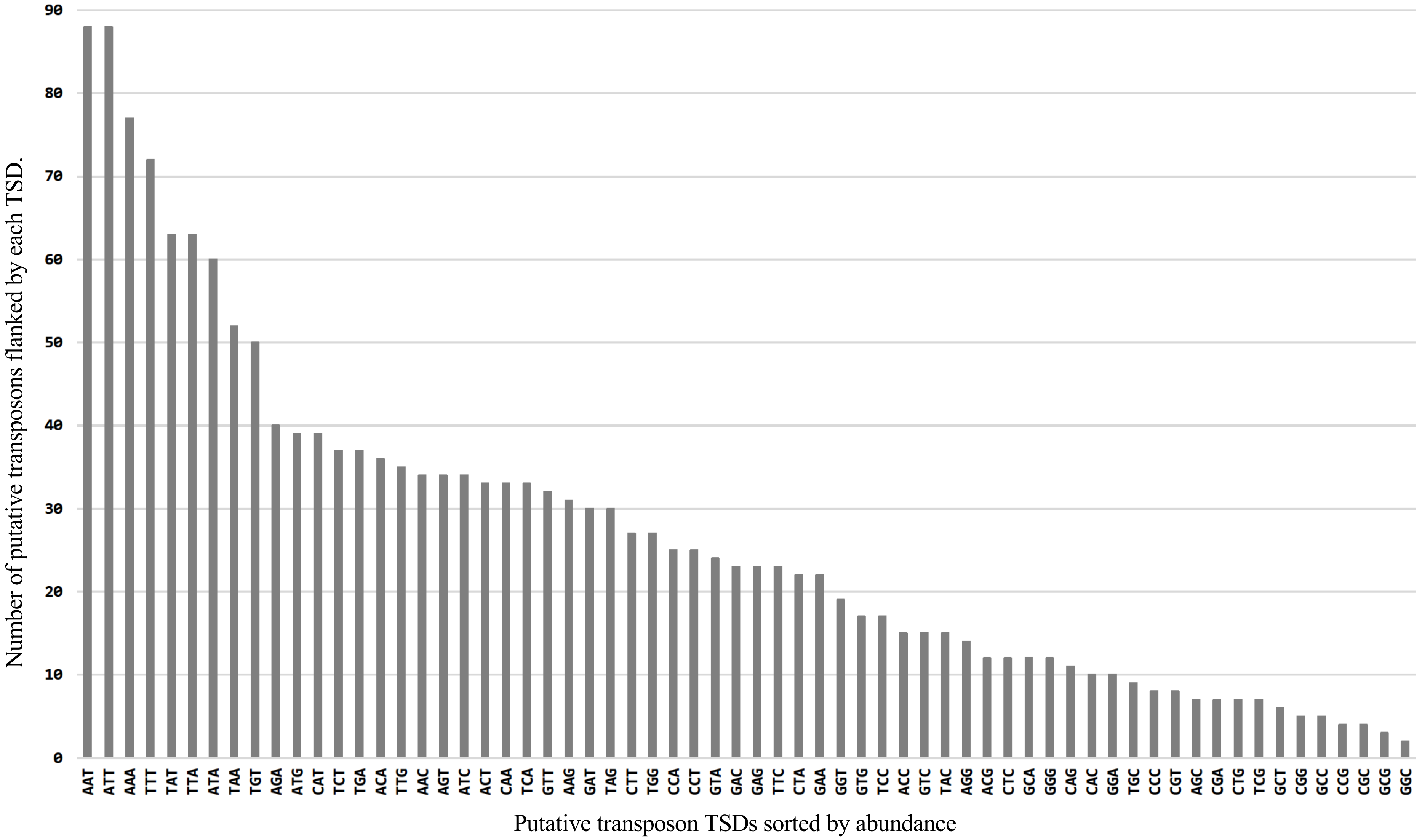

Supplemental Figure S5. Target site duplication (TSD) frequencies of putative *LsC1* family transposons in the *cv*. Salinas lettuce genome. All TSD tri-nucleotides flanking the 1,714 putative *LsC1* family transposons (see Supplemental Figure S4) in the *cv*. Salinas lettuce genome (GenBank RefSeq GCF_002870075) were counted and sorted according to their abundance. The 64 TSDs are listed on the X-axis in decreasing abundance; the number of putative transposons flanked by each TSD is shown on the Y-axis.

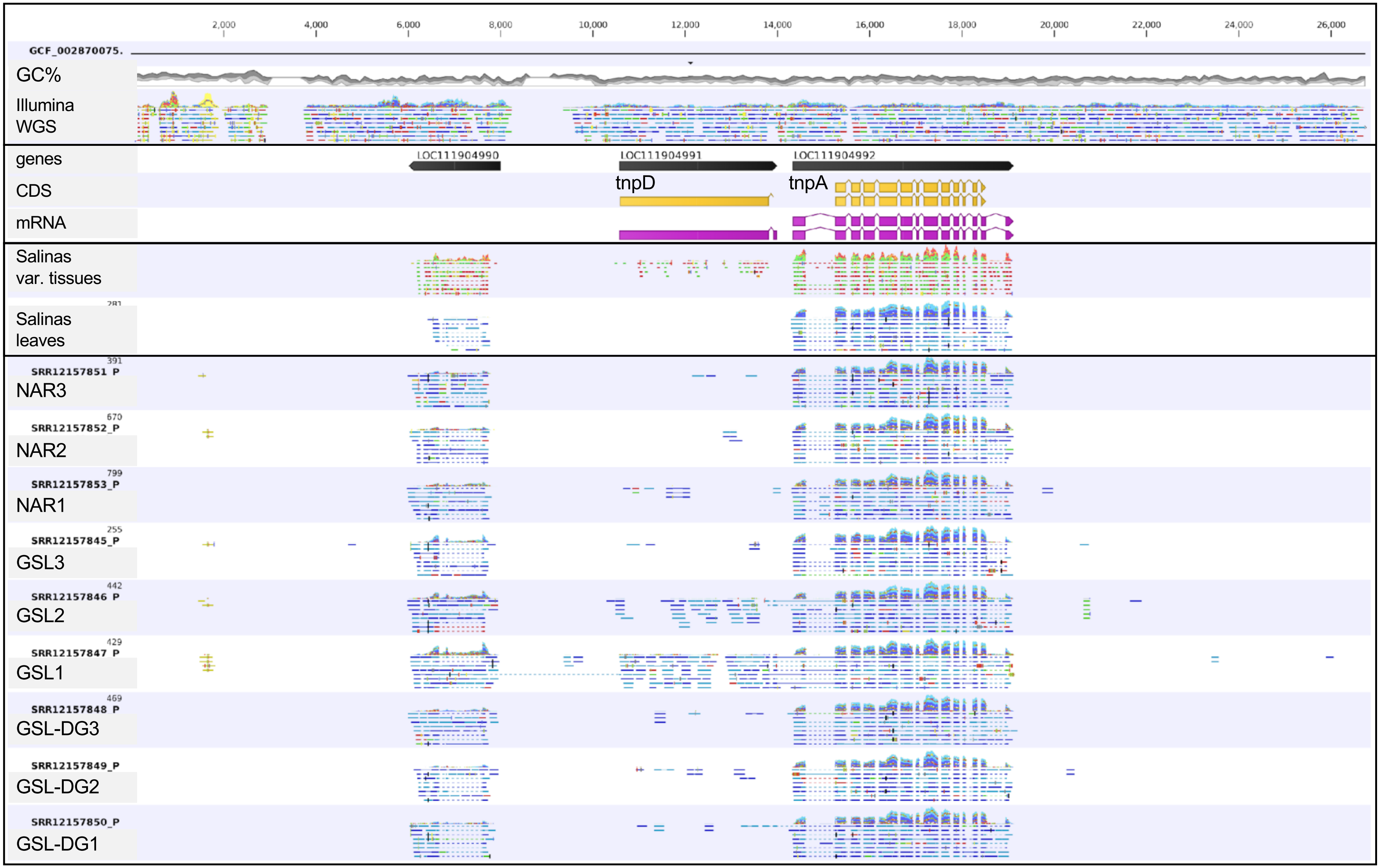

Supplemental Figure S6. Expression from a putative *tnpD-tnpA* locus in *cv*. Salinas, NAR, GSL, and GSL-DG shows contiguous transcription across the two putative genes in two of three GSL samples. Shown is a putative lettuce *tnpD-tnpA* expressed complex (loci LOC111904991 and LOC111904992 from V7 RefSeq) identified by bioinformatics analysis, from 3-3 biological replicates of NAR, GSL-DG, and GSL, in addition to two *cv*. Salinas transcriptome sets: various tissues (SRR080725 & SRR085107 Matvienko et al., 2013) and leaves (SRR9659238–SRR9659240 Park, Shi and Mou, 2020). Genome annotation and RNA-seq read mappings were visualized with CLC Genomics Workbench Track Viewer. Note the contiguous transcription between *tnpD* and *tnpA* for samples GSL1 and GSL2.
